# A National Multicenter Study on Initial Antiviral Treatment Preferences on Chronic Hepatitis B: Entecavir Versus Tenofovir Disoproxil Fumarate

**DOI:** 10.5152/tjg.2025.24741

**Published:** 2025-10-23

**Authors:** Tansu Yamazhan, Esra Zerdali, Yusuf Önlen, Selma Tosun, Özgür Günal, Ayşe Batırel, İmran Hasanoğlu, Tuba Turunç, Umay Balcı, Sibel Yıldız Kaya, Oğuz Karabay, İlknur Esen Yıldız, Lütfiye Nilsun Altunal, Hacer Deniz Özkaya, Selçuk Kaya, Ayşe İnci, Sevil Alkan, Dilek Sevgi Yıldız, Tayibe Bal, Esma Aslıhan Aydemir, Nurullah Eser, Serhat Uysal, Oğuzhan Acet, Fehmi Tabak, Rahmet Güner

**Affiliations:** 1 Department of Infectious Diseases and Clinical Microbiology, Ege University Faculty of Medicine, Izmir, Türkiye; 2 Department of Infectious Diseases and Clinical Microbiology, University of Health Sciences Turkey, Haseki Training and Research Hospital, İstanbul, Türkiye; 3 Department of Infectious Diseases and Clinical Microbiology, Hatay Mustafa Kemal University Faculty of Medicine, Hatay, Türkiye; 4 Department of Infectious Diseases and Clinical Microbiology, İzmir City Hospital, İzmir, Türkiye; 5 Department of Infectious Diseases and Clinical Microbiology, Samsun University Faculty of Medicine, Samsun, Türkiye; 6 Department of Infectious Diseases and Clinical Microbiology, İstanbul Lutfi Kırdar Kartal Training and Research Hospital, İstanbul, Türkiye; 7 Department of Infectious Diseases and Clinical Microbiology, Ankara Yıldırım Beyazıt Faculty of Medicine, Ankara, Türkiye; 8 Department of Infectious Diseases and Clinical Microbiology, Adana Health Sciences University Faculty of Medicine, Adana, Türkiye; 9 Department of Infectious Diseases and Clinical Microbiology, University of Health Sciences Turkey, Antalya Training and Research Hospital, Antalya, Türkiye; 10 Department of Infectious Diseases and Clinical Microbiology, İstanbul University Cerrahpaşa Medical Faculty, İstanbul, Türkiye; 11 Department of Infectious Diseases and Clinical Microbiology, Sakarya University Medical Faculty, Sakarya, Türkiye; 12 Department of Infectious Diseases and Clinical Microbiology, Recep Tayyip Erdoğan University Faculty of Medicine, Rize, Türkiye; 13 Department of Infectious Diseases and Clinical Microbiology, University of Health Sciences, Ümraniye Training and Research Hospital, İstanbul, Türkiye; 14 Department of Infectious Diseases and Clinical Microbiology, Bakırçay University, Çiğli Training and Research Hospital, İzmir, Türkiye; 15 Department of Infectious Diseases and Clinical Microbiology, Çanakkale Onsekiz Mart University Faculty of Medicine, Çanakkale, Türkiye; 16 Department of Infectious Diseases and Clinical Microbiology, University of Health Sciences, İstanbul Training and Research Hospital, İstanbul, Türkiye; 17 Department of Infectious Diseases and Clinical Microbiology, University of Health Sciences, Şişli Hamidiye Etfal Training and Research Hospital, İstanbul, Türkiye; 18 Department of Infectious Diseases and Clinical Microbiology, Bolu Abant Izzet Baysal Faculty of Medicine, Bolu, Türkiye; 19 Department of Infectious Diseases and Clinical Microbiology, Fırat University Faculty of Medicine Elazığ, Türkiye

**Keywords:** Chronic hepatitis B, entecavir, tenofovir disoproxil fumarate

## Abstract

**Background/Aims:**

Selecting the initial antiviral regimen for chronic hepatitis B (CHB) requires balancing patients’ comorbidities and long-term safety. This study examines the differences in patient and disease-related factors that guide clinicians to prescribe either entecavir (ETV) or tenofovir disoproxil fumarate (TDF) as the initial treatment.

**Materials and Methods:**

The study included treatment-naïve CHB patients aged 18 or older who had been diagnosed for at least 1 year since 2010 and initiated on antiviral therapy. The data included variables such as age, gender, body mass index (BMI), comorbidities, liver disease activity, biopsy results, cirrhosis, hepatic steatosis, hepatitis B e-antigen status, hepatitis B virus DNA levels, triglycerides, cholesterol, renal function, and baseline bone mineral density (BMD), which were assessed by dual-energy x-ray absorptiometry (DEXA).

**Results:**

Among 2259 patients (61.6% male), 1270 patients (56.22%) received TDF, while 989 patients (43.78%) received ETV as first-line therapy. The TDF was more commonly prescribed to patients with a lower BMI (median 25.7 vs. 26.2, *P* = .001) and lower baseline creatinine (0.75 vs. 0.80 for ETV, *P* < .001). Clinicians preferred ETV among patients with an estimated glomerular filtration rate (eGFR) < 60 (n = 36), (*P* < .001). The BMD was evaluated in 365 patients (16.3%). The DEXA scans were performed for 116 patients (11.8%) in the ETV group and 249 patients (19.8%) in the TDF group (*P* < .001).

**Conclusions:**

This national multicenter study emphasizes that patient-related factors, including gender, age, baseline renal function, and liver disease severity, significantly influence the choice of first-line antiviral therapy for CHB, often outweighing disease-specific factors.

**Main Points**

- Baseline kidney function tests are critical in guiding the choice of antiviral medication for chronic hepatitis B.
- Determining baseline bone mineral density, although not commonly performed, impacts the preference for tenofovir disoproxil fumarate, as it possesses a greater risk of reducing bone density over time compared to entecavir.
- Clinicians prioritize factors like age, gender, renal function, and liver disease severity over other comorbidities when selecting antiviral therapy.

## Introduction

Today, no available medications for chronic hepatitis B (CHB) can completely eradicate the virus. Therefore, the primary goal of effective therapy is to achieve a functional cure, which often requires long-term or even lifelong treatment strategies.^1^^-^^3^ Nucleoside and nucleotide analogues, such as entecavir (ETV) and tenofovir disoproxil fumarate (TDF) or tenofovir alafenamide (TAF), are the mainstay of CHB treatment due to their potent antiviral efficacy, high resistance barrier, and safety.^4^

National and international guidelines spotlight the importance of evaluating patient-related factors and potential long-term side effects when selecting initial antiviral therapy.^5^^-^^7^ Key considerations include age, gender, pregnancy plans, and the presence of comorbidities such as renal and bone diseases. This study aims to investigate whether there are statistically significant differences in patient or disease-related factors that affect clinical decision making to prescribe TDF or ETV as the initial treatment in patients with chronic hepatitis B virus (HBV).

## Materials and Methods

### Study Population and Data Collection

This study is a part of the “Chronic Hepatitis B Patient Registry Study: National Multicenter Retrospective Study.” Patient data were collected by researchers at multiple centers (Adana, Ankara, Antalya, Bolu, Çanakkale, Elazığ, Hatay, İstanbul, İzmir, Rize, Sakarya, Samsun) using an electronic patient follow-up form between August 2021 and October 2023. Patients included in the study were aged 18 years or older, were treatment-naïve, and had been diagnosed with chronic HBV for at least 1 year since 2010. They were initiated on antiviral therapy with either ETV (0.5 mg/day) or TDF (245 mg/day). Patients were excluded from the study if antiviral agents were started as prophylaxis in the context of immunosuppression, had co-infections (Human Immunodeficiency Virus, Hepatitis C Virus, or Hepatitis D Virus), and/or had concomitant liver diseases such as alcoholic or autoimmune liver disease.

Patients were eligible for antiviral therapy with nucleos(t)ide analogues if they met one or more of the following criteria: the presence of cirrhosis with detectable HBV DNA levels; liver fibrosis stage ≥2 or a modified histological activity index (HAI) score >6 (Ishak system) with HBV DNA > 2000 IU/mL and ALT above the upper limit of normal (ULN); HBV DNA > 20 000 IU/mL and alanine aminotransferase (ALT) > 2 × ULN; or the presence of extrahepatic manifestations.^6^^-^^9^

The data collected for this study included patient demographics, such as age, gender, age at diagnosis, and age at therapy initiation. Information about comorbidities was also recorded, including renal failure, heart disease, history of myocardial infarction, hypertension, thyroid disorders, and diabetes. Liver disease parameters, such as ALT, aspartate transaminase, total bilirubin, platelet count, prothrombin time, liver biopsy findings (fibrosis stage and HAI scores), cirrhosis, and hepatic steatosis, were included. Viral markers, such as hepatitis B e-antigen (HBeAg) status and baseline HBV DNA levels, were documented as well. Additionally, host factors, including baseline creatinine levels, estimated glomerular filtration rate (eGFR), cholesterol, triglyceride levels, and bone mineral density (BMD) determined via dual-energy x-ray absorptiometry (DEXA), were evaluated. Comparative analyses between the 2 antiviral drugs included host factors such as gender, age at treatment initiation, body mass index (BMI), presence of comorbidities, baseline creatinine levels, triglycerides, cholesterol, eGFR, and BMD results.

### Statistical Analysis

The Pearson chi-square test was applied to identify differences in parameters influencing the initial choice of either ETV or TDF, while the Mann–Whitney *U* test was used for the analysis of numerical data. A *P*-value of less than .05 was considered statistically significant. All statistical analyses were conducted using SPSS, version 21 (IBM SPSS Corp.; Armonk, NY, USA). The study received ethical approval from the Ege University Clinical Research Ethics Committee on July 6, 2021, under decision number A-83. Informed consent was obtained from all patients.

## Results

This study included data from 31 centers located in 21 different provinces across Türkiye. The median follow-up period for patients was 72.3 months (min-max: 15-156). A total of 2259 patients were evaluated, comprising 1391 males (61.6%) and 868 females (38.4%). Among these participants, 1270 patients (56.22%) were initiated on therapy with TDF, while 989 patients (43.78%) had ETV as their first-line antiviral medication. Viral factors such as HBeAg status and HBV DNA levels (measured using the Cobas® 6800 system, Roche Molecular Diagnostics, Switzerland) were analyzed and are summarized in Table 1. No statistically significant differences were observed between the ETV and TDF groups in terms of comorbid conditions including chronic renal failure, cardiac disease, hypertension, thyroid disease, and diabetes mellitus (*P* > .05 for all). Liver disease-related parameters such as the presence of cirrhosis, baseline ALT levels, HAI, fibrosis scores, and hepatosteatosis also showed no significant differences between the treatment groups (*P* > .05). Similarly, baseline lipid profiles (cholesterol and triglycerides) were comparable, with no statistically significant differences observed (*P* = .057 and *P* = .97, respectively).

The ETV was more commonly prescribed to male patients, whereas TDF was more frequently chosen for female patients (*P* = .005). The mean age and standard deviation (SD) at diagnosis and treatment initiation for patients receiving ETV were 40 ± 13 and 44 ± 13 years, respectively, while for those receiving TDF, it was 36 ± 13 and 40 ± 12 years, respectively. There was a statistically significant difference between these (*P* < .001), indicating that TDF was more often preferred for younger patients at both diagnosis and treatment initiation. A similar trend was observed for BMI. The TDF was more commonly selected as the first-line therapy for patients with a lower BMI, with a median of 25.7 (18.52-48.28) compared to 26.2 for ETV (15.57-55.56) (*P* = .001).

**Figure 1.**
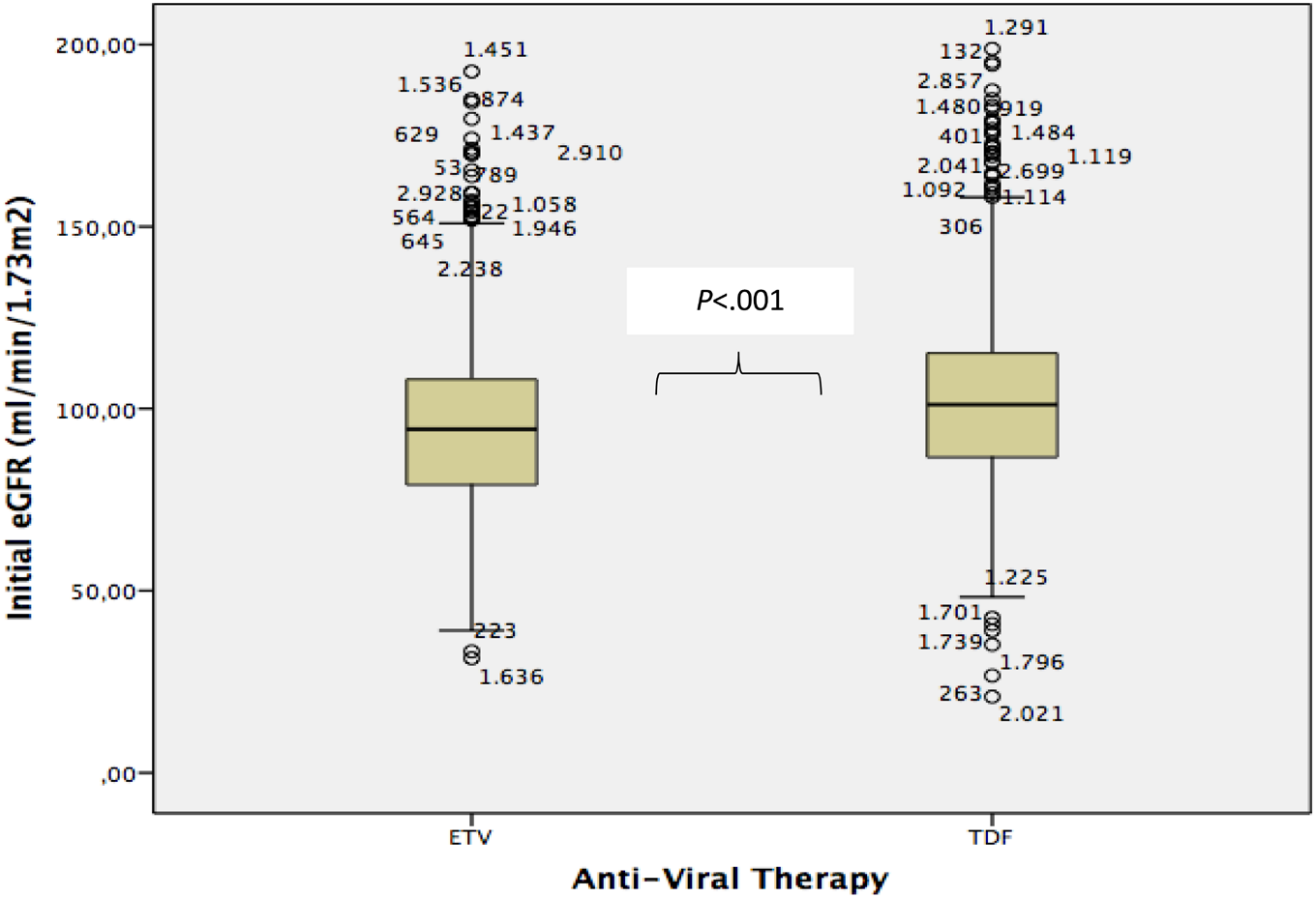
The graph shows the difference between eGFR results at the time of starting antiviral treatments.

Baseline renal functions also played a part in the decision-making process for the antiviral drug choice. The median baseline creatinine levels (min-max) were 0.75 (0.10-5.60) for TDF and 0.80 (0.10-8.00) for ETV, with ETV being preferred in patients with higher creatinine levels (*P* < .001). A similar pattern was observed with eGFR. The median eGFR (mL/min/1.73 m^2^) for the ETV group was 94.37 (min-max: 31.47-179.61), while for the TDF group, it was 101.12 (min-max: 20.91-179.44). Although the number of patients with baseline eGFR < 60 (n = 36) was not large, physicians significantly preferred ETV as the initial drug choice for these patients upon re-evaluation (*P* < .001). The relationship between eGFR values and drug selection is illustrated in Figure 1.

The BMD was determined in 365 patients (16.3%) before the initiation of treatment. The DEXA tests were performed in 116 patients (11.8%) who started ETV and 249 patients (19.8%) who started TDF. A higher percentage of patients were commenced on TDF among patients who had available DEXA results, which showed statistical significance (*P* < .001).

## Discussion

In Türkiye, 1 in 3 individuals over the age of 18 has been exposed to HBV, rendering this a significant cause of liver transplantation nationwide.^10^^-^^13^ The incidence and prevalence of the disease have decreased owing to the national vaccination program, which has immunized newborns against HBV since 1998, as well as the use of antivirals for chronic cases.^14^ Potent antivirals such as ETV, introduced in 2007, and TDF, introduced in 2008, have been included in the Communique on Healthcare Practices for the treatment of chronic HBV in Türkiye and can be prescribed by Gastroenterology or Infectious Diseases specialists with appropriate indications. The TAF was conditionally introduced as a second-line treatment for HBV in 2018.^8^ The use or switch to TAF is authorized in cases of proven renal or bone pathologies. However, in 2020, this condition was removed, and both forms of tenofovir, along with ETV, were included in first-line treatment for chronic HBV with reimbursement. Therefore, data from patients using TAF were not included in the study due to the small number of cases.

In this study, TDF was more commonly selected as the first-line treatment for CHB patients compared to ETV. Gender was a significant factor influencing antiviral selection, with TDF being prescribed more frequently to women. This trend is primarily attributed to the fact that TDF has been shown to be safe and effective in reducing serum HBV DNA concentrations to low or undetectable levels in pregnant women. When combined with passive and active immunization, TDF also reduces the risk of intrauterine and perinatal transmission of HBV.^15^^-^^19^ In the study group, TDF was significantly more likely to be selected for younger patients at the time of diagnosis and treatment initiation compared to ETV. This preference may reflect clinical inclination to choose a safer antiviral agent, particularly considering the potential for pregnancy in younger female patients. However, regardless of gender, TDF was also statistically more preferred in younger patients at the time of diagnosis and treatment initiation compared to ETV. This finding is in line with clinical practice guidelines, which recommend TAF or ETV over TDF for elderly patients and those with existing decreased BMD or renal conditions.^20^ High TDF plasma trough concentrations and patient age have been identified as independent risk factors for drug-induced kidney and bone toxicity. HIV and hepatitis B co-infection are also frequently observed in the country, and careful attention should be paid to these adverse effects in this population.^21^ Particularly, HIV-positive women with low body weight are particularly vulnerable to elevated TDF plasma trough concentrations, which increases their risk of developing drug-related complications. Gervasoni et al^22^ demonstrated that HIV-positive women with low body weight are more susceptible to toxic side effects associated with high plasma concentrations of TDF. Hence, plasma monitoring is recommended to mitigate potential side effects of TDF use in women. Despite these concerns, the preference for TDF in the study may be driven by contemporary research on its safety profile, its lower resistance rates compared to ETV, and the likelihood of infection with lamivudine-resistant HBV strains.

In determining the choice of antiviral therapy between ETV and TDF, no significant difference was observed based on patient comorbidities. Notably, the absence of a difference in drug selection among patients diagnosed with chronic kidney disease or reporting having had renal issues was a surprising finding. However, in the patient group, differences in baseline renal function tests at the initiation of therapy created a statistically significant distinction in the choice between TDF and ETV. This suggests that clinicians may prioritize current laboratory test results over patient anamnesis when making clinical decisions.

Renal side effects are frequently emphasized in numerous clinical guidelines as a critical adverse effect requiring close monitoring during the long-term use of antivirals for HBV treatment. Long-term drug accumulation in renal proximal tubules can lead to tubular damage and a decline in eGFR.^23^ Among antivirals, TDF is most associated with tubular damage due to its high plasma concentration. Liu et al^23^ conducted a meta-analysis that included studies focusing on renal function indices, such as creatinine and eGFR. After reviewing 16 studies involving 4278 adults receiving treatment for CHB with ETV, TDF, or TAF, they found that TDF had a more significant adverse impact on renal function compared to TAF or ETV. Given the necessity for close monitoring of renal side effects during the long-term use of TDF, as highlighted in treatment guidelines, clinicians in ther study group preferred ETV for patients with high baseline creatinine, lower eGFR, or an eGFR <60 mL/min/1.73 m^2^.

Another long-term side effect of TDF is osteoporosis and the associated risk of bone fractures due to a reduction in BMD. This was first demonstrated in randomized controlled clinical trials involving HIV positive patients, where those treated with TDF experienced cases of osteomalacia and bone fractures linked to decreased BMD.^24^^-^^26^ A study conducted on patients with chronic HBV using TDF for extended periods reported a decrease in BMD of less than 2%.^27^ In a study by Gill et al,^28^ demographic data, serum bone biochemical tests, and BMD measurements using DEXA were conducted in patients with chronic HBV treated with TDF. Fracture Risk Assessment Tool (FRAX) scores were calculated before and after the DEXA tests. The study found that patients with CHB treated with TDF exhibited reduced BMD, though the reduction was limited to 1e anatomical site. In a meta-analysis by Liu et al,^23^ current antivirals were compared in terms of bone-related side effects. The analysis revealed that during long-term treatment, such as at the 60-month mark, the decrease in BMD due to hypophosphatemia associated with TDF use was significantly greater in patients using TDF compared to those using ETV. In this study, a limited number of patients underwent DEXA tests to determine BMD. However, it is noteworthy that, although in a small percentage, more DEXA tests were performed in the group scheduled to start TDF, possibly due to concerns about bone-related side effects. The low frequency of DEXA testing in the patient group, which consists of individuals who began treatment after 2010, may be attributed to the limited available knowledge and experience regarding antiviral side effects at that time.

The order in which drugs for chronic HBV treatment were introduced in the country, national reimbursement policies, and international and national hepatitis treatment guidelines are key factors that guide the choice for initial antiviral selection. A major limitation of this study is the inability to assess the extent to which physicians consider these factors when selecting antiviral drugs. In a web-based survey conducted in Türkiye on drug choice for CHB involving both patients and physicians, it was found that while patients prioritize the efficacy of the drug, physicians prioritize efficacy alongside the risk of renal failure.^29^ This observation aligns with the findings of the study. Another important limitation is the lack of follow-up data on the long-term effects of antiviral drugs. Additionally, the inability to obtain pre-treatment BMD data for every patient has made it difficult to comprehensively evaluate this issue and can be considered another limiting factor.

In conclusion, this study represents a significant national multicenter collaboration with a comprehensive patient cohort, shedding light on the key factors considered by infectious diseases and hepatology specialists when initiating treatment for patients with CHB. Patient-specific variables, such as gender, age at diagnosis and treatment initiation, baseline renal function, HBeAg status and the stage of the liver disease, were found to be more guiding than the disease itself in determining the choice of antiviral therapy.

## Figures and Tables

**Table 1.** Demographic Findings, Comorbidities, Viral and Hepatic Findings, and Baseline Biochemical Values of CHB Patients who Will Be Started on Medication

	Entecavir (n = 989)	Tenofovir Disoproxil Fumarate (n = 1270)	*P*
Male n (%)	641 (64.8)	750 (59.1)	.005
Female n (%)	348 (35.5)	520 (40.9)	
Age at diagnosis, median, (min-max)	40 (18-85)	36 (18-83)	<.001
Age at treatment, median, (min-max)	44 (18-88)	40 (18-83)	<.001
BMI (kg/m^2^), median, (min-max)	26.2 (15.57-55.56)	25.7 (18.52-48.28)	.001
Comorbidities, n (%)			
Chronic renal failure	19 (1.9)	13 (1)	.07
Cardiac disease	66 (6.7)	71 (5.6)	.29
Hypertension	171 (17.4)	188 (14.8)	.10
Thyroid disease	22 (2.2)	43 (3.4)	.10
Diabetes mellitus	128 (13)	148 (11.7)	.35
Initial parameters of the virus			
HBeAg positivity n (%)	221 (23.6)	274 (23.3)	.89
HBV-DNA (log10 IU/mL), median, (min-max)	5.61 (3.30-9.98)	5.67 (3.30-12.49)	.68
Disease activity in the liver			
Presence of cirrhosis, n (%)	55 (5.6)	71 (5.6)	.95
Baseline ALT, median, (min-max)	47 (5-3610)	46 (6-4680)	.95
HAI, median, (min-max)	7 (2-15)	7 (1-18)	.99
Fibrosis, median, (min-max)	2 (0-6)	2 (0-6)	.21
Presence of hepatosteatosis, n (%)	216 (22)	293 (23.3)	.47
Baseline biochemical parameters			
Creatinine, median, (min-max)	0.80 (0.10-5.60)	0.75 (0.10-8.00)	<.001
Cholesterol, median, (min-max)	187 (77-474)	179 (84-402)	.057
Triglycerides, median, (min-max)	100 (23-399)	101 (12.4-568)	.97
eGFR, median, (min-max)	94.37 (31.47-179.61)	101.12 (20.91-179.44)	<.001
eGFR < 60, n (%)	38 (4.7)	18 (1.7)	<.001
DEXA test performed n (%)	116 (11.8)	249 (19.8)	<.001

ALT, alanine aminotransferase; BMI, body mass index; DEXA, dual-energy x-ray absorptiometry; eGFR, estimated glomerular filtration rate; HAI, histological activity index; Max, maximum; Min, minimum.

## Data Availability

The data that support the findings of this study are available on request from the corresponding author.
